# Influence of Insertion Torques on the Surface Integrity in Different Dental Implants: An Ex Vivo Descriptive Study

**DOI:** 10.3390/ma16062330

**Published:** 2023-03-14

**Authors:** Erika Brancacci, Susana García González, Andrea Galve-Huertas, Aida Bennani, Federico Hernández Alfaro, Samir Aboul-Hosn Centenero

**Affiliations:** 1 Department of Oral & Maxillofacial Surgery, Universitat Internacional de Catalunya, 08195 Sant Cugat del Vàlles, Spain; 2 Department of Dentistry, Universitat Internacional de Catalunya, 08195 Sant Cugat del Vallès, Spain

**Keywords:** implant surface, implant connection, topographic change, insertion torque

## Abstract

Background: The primary objective of this ex vivo study was to assess the influence of increasing insertion torques on three types of dental implants and possible alterations of their microgeometry after the application of three different torque intensities. Methods: 27 implants of 3 different implant brands (Groups A, B and C) were placed in cow ribs using 30 Ncm, 45 Ncm and 55 Ncm insertion torques. The implants were subsequently removed using trephine burs, and SEM analysis was carried out in order to detect implant surface and connection changes, as compared to the implant controls. Results: Surface deformations were predominantly observed on the third apical part of the implants. The alterations presented with increasing insertion torques, with 45 Ncm being the threshold value. Prosthetic connections were also compromised. Conclusions: The changes sustained by the implants were proportional to the insertion torque they were subjected to; 45 Ncm and greater insertion torques resulted in more consistent damage, both on the implant surface and the implant connection.

## 1. Introduction

Primary stability is recognized as an essential criterion for obtaining future osseointegration. It can be defined as the mechanical stability obtained immediately after the insertion of a dental implant, or in simpler words, the bone–implant fixation force. It is a measure of the quality of anchoring an implant in the alveolar bone, and it should be measured immediately after insertion since stability levels may vary over time due to bone remodeling at the bone–implant interface [1].

During implant placement, the clinician perceives the stability of the implants according to the rotational resistance produced when inserting them [2]. Various authors [3,4,5] suggest that a high insertion torque (IT) in implants is desirable to improve osseointegration phenomena since torque has a directly proportional relationship with primary stability [6] since the gap between the implant surface and the surrounding bone is reduced, thus decreasing the possible micromotion [5,7].

However, this procedure is associated with the possible appearance of bone overcompression and, thus, bone necrosis, which could cause the failure of osseointegration [8]. Moreover, overload or irregular distribution of forces due to the high torque insertion can produce alterations and deformations in the different structures of the implants [9]. In fact, surface changes have been observed at the level of the platform, causing loosening of the retaining screw, damage to the implant driver and wear of the internal threads of the implants [10].

The design of dental implants and their surfaces are important factors to consider from the point of view of load transmission, bone remodeling and maturation. When choosing an implant system, the clinician must take into account the behavior of the roughness of the surfaces of the chosen implants when subjected to insertion torque forces. In fact, this factor will not only have a great impact on the stability of the implant when inserted in the bone but also on the predictability of the behavior of the osteoblasts, and consequently in the clinical success of the implant treatment, especially in the long term and in situations of immediate or early loading [11].

Even if the high torques reduce the risk of micromotion at the bone–implant interface and allow a greater degree of success in immediate loading, these implants can have external or internal morphological changes during torsion when inserted into bone [12]. If we apply more insertion torque than the maximum allowed, alterations in the bone structure may occur [13]. Additionally, when placing dental implants with a high insertion torque of more than 50 Ncm [14], the transmission of large compressive stresses to the neighboring bone may occur [15,16,17], which can compromise the success of osseointegration. Accordingly, it is necessary to establish correct implant torque protocols to avoid bone and/or implant damage, with the consequent failure of prosthetic rehabilitation [10,13].

There is a wealth of bibliographic documentation on the behavior of implant surfaces prior to insertion; some studies are based on finite element analysis, which could be difficult to extrapolate to clinical practice [18]. Additionally, when we consider the effects of friction forces during and after the insertion into the bone, and the impact on the roughness of the endosteal surfaces, we found few studies that examine this behavior [19]. To the best of the authors’ knowledge, this is the first ex vivo study that analyzes the behavior of different implant surfaces submitted to different implant insertion torques.

The aim of this descriptive observational study is to analyze the topography of the implant surface inserted in the bovine rib when undergone through the same procedure at different insertion torques, assessing the results of the possible involvement of the macro-geometry micro-roughness and prosthetic connection.

## 2. Materials and Methods

### 2.1. Study Design

The present study was designed as an in vitro study in which 30 implants of 3 different brands were tested (10 implants per implant brand). One implant of each brand was used as a control and nine as a test:

- Group A;
- Group B;
- Group C.

**Figure 1:**
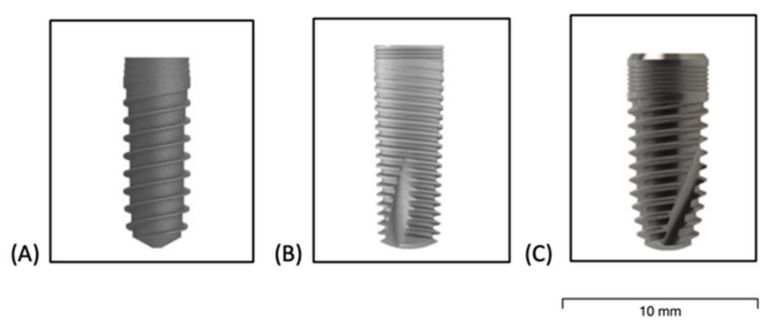
Implant models of the 3 brands: Group (**A**): 4.0 × 10 mm; Group (**B**): 3.75 × 10 mm; Group (**C**): 4.20 × 10 mm.

Group A:

Group A implant used in this study was a grade 5 Alloy Al6V4, self-tapping conical implant with internal connection and platform switching. The type of surface was a thermochemical treatment consisting of an alkaline immersion followed by a heat treatment (Figure 1A).

Group B:

Group B is a conical implant with a conical connection with a surface comprised of grade 23 titanium alloy Ti-6Al-4V-ELI (extra-low interstitial). The surface treatment of this group was a combination of sandblasting and acid etching (Figure 1B).

Group C:

Group C is a grade 4 titanium (cold forming method) conical implant with an internal hexagonal connection. It presents a surface treatment consisting of sandblasting and double acid etching, creating a surface with a roughness of 1.4 μm (Figure 1C).

### 2.2. Sample Size Calculation

Sample size calculation was performed in order to assess the correlation between the insertion torque and the changes in the implant surface. The implants were placed with three different insertion torques 30 Ncm, 45 Ncm and 55 Ncm. In order to evaluate the correlation between the IT and the topographic changes in the implant surface, using an alpha risk of 5% and a beta risk of 20% and assuming that the correlation can be at least 0.5 and without loss to follow-up, the sample size calculation yielded 30 experiments.

### 2.3. Pre-Surgical Protocol

Before subjecting any implant to insertion torque, the surfaces of the three control implants were analyzed with the SEM; this consisted of the starting point of these surfaces. The implant surfaces were examined using 50× magnification.

### 2.4. Surgical Protocol

**Figure 2:**
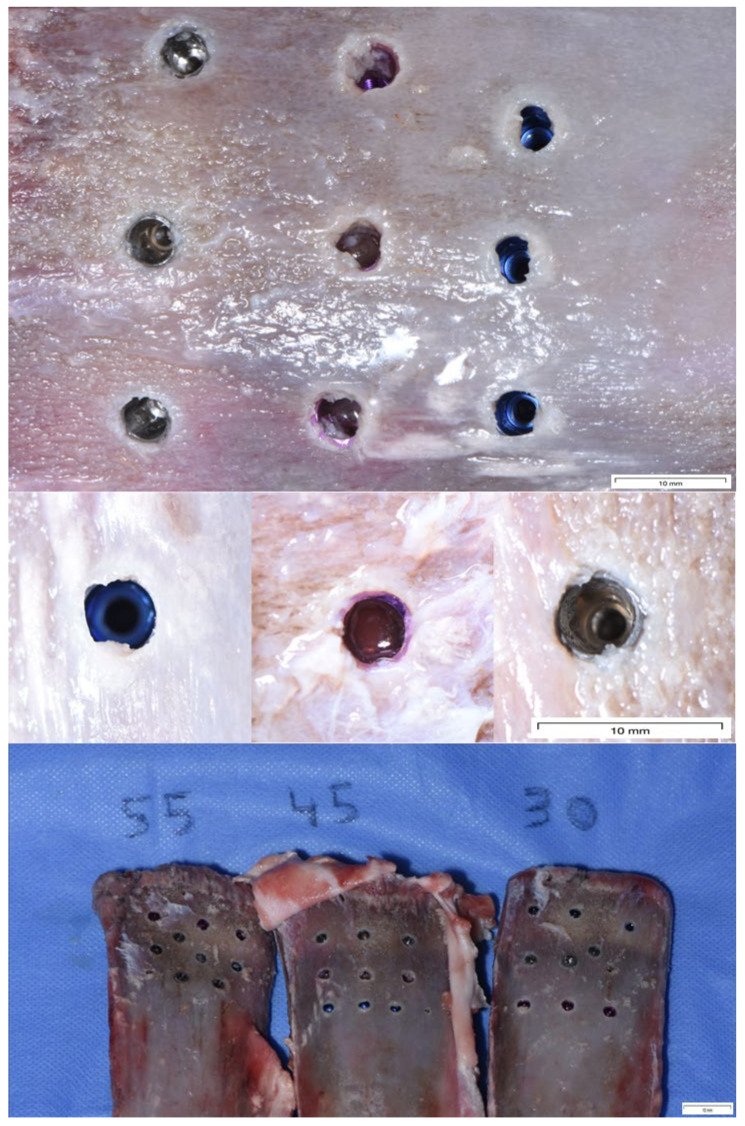
Picture of the 27 implants placed in 3 porcine ribs in which 3 implants for each brand were placed with the same insertion torque. The first rib on the left of the image was inserted with 9 implants of the 3 brands at 55 Ncm torque, the middle rib with 9 implants of the 3 brands at 45 Ncm, and the right rib with 9 implants of the 3 brands at 30 Ncm.

A total of 3 porcine ribs (provided by MAXYLAR BONES, SL, Barcelona, Spain) were used for this experiment. The first rib was used to place 9 implants with 30 Ncm, the second rib to place 9 implants with 45 Ncm, and the third rib to place 9 implants with 55 Ncm (Figure 2). In total, 27 implants were placed with different insertion torques. The same experts were responsible for implant placement, following a strict protocol recommended by the implant system manufacturers.

Three implants of each brand were placed in the different ribs so that the bone quality of the ribs did not influence the results. All implants were placed with a motor. The three experts (SA, SG, AG) always placed the same implant brand. The ribs were of type 2 bone quality, according to Lekholm and Zarb. Once the implants were placed, they were removed from the rib, using a trephine bur, for their subsequent microscopic observation.

Once the implants were removed from the trephines, they were placed in beakers and cleaned of organic matter using 30% hydrochloric acid at room temperature for ten minutes, eliminating the organic part without influencing the implant surface. Furthermore, the surfaces that seemed to have topographic defects were exposed to oxygen plasma. In the event of doubt of biological contamination, hydrochloric acid was used to eliminate the remanent organic matter. Finally, the implants were stored in plastic bags marking their respective insertion torques.

### 2.5. Microscopic Analysis

This study was carried out with a Jeol JSM 5410 (Tokyo, Japan) (SEM) located at the International University of Catalonia (UIC). It was not essential to metalize the implants prior to SEM observations since they ensure sufficient conductivity and adequate image quality. Digital images were acquired through INCA microanalysis suite 4.04 software; Oxford Instruments, Abingdon, UK). In the study, images were first taken at low magnifications (50 × magnifications) of both the vertices of the internal hexagon and the threads, then observations were made at higher magnifications (2k or higher). First, the evaluation zones (implant connection and apical third) of the three control implants were analyzed to assess the starting point and to be able to compare with what we would observe after subjecting the implants to IT.

**Figure 3:**
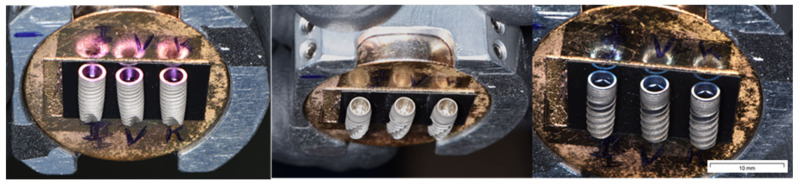
Implants placed in the sample holder of the SEM. Each implant brand was analyzed. The implants subjected to the highest torque presented visually detectable surface deformations at the level of the apical third.

The sample was fixed with two bolts on a sample holder parallel to the floor, and after a few seconds, a vacuum was created in the pre-chamber of the microscope. After this time, the preparation was placed inside the vacuum chamber for its subsequent analysis. The measurement system consisted of analyzing each implant of the same insertion torque of each brand in the same order (Figure 3).

## 3. Results

The SEM analysis showed that after an application of 30 Ncm, only small marks were observed on Group A implant surface and connection, while Group B and Group C implants showed more surface alterations. Many of the minor surface features were less noticeable in the apical third, indicating that they may have been removed. The apical cutting threads were subjected to a large level of tension, and the damage was concentrated in this area for the three different brands.

The same scenario was shown at the level of the connection of the implants. It was observed that from the application of 45 Ncm and 55 Ncm, deformations were observed at the level of the implant connection. As we can see in the SEM images, the higher the torque of insertion was, the more substantial the alterations were.

**Figure 4:**
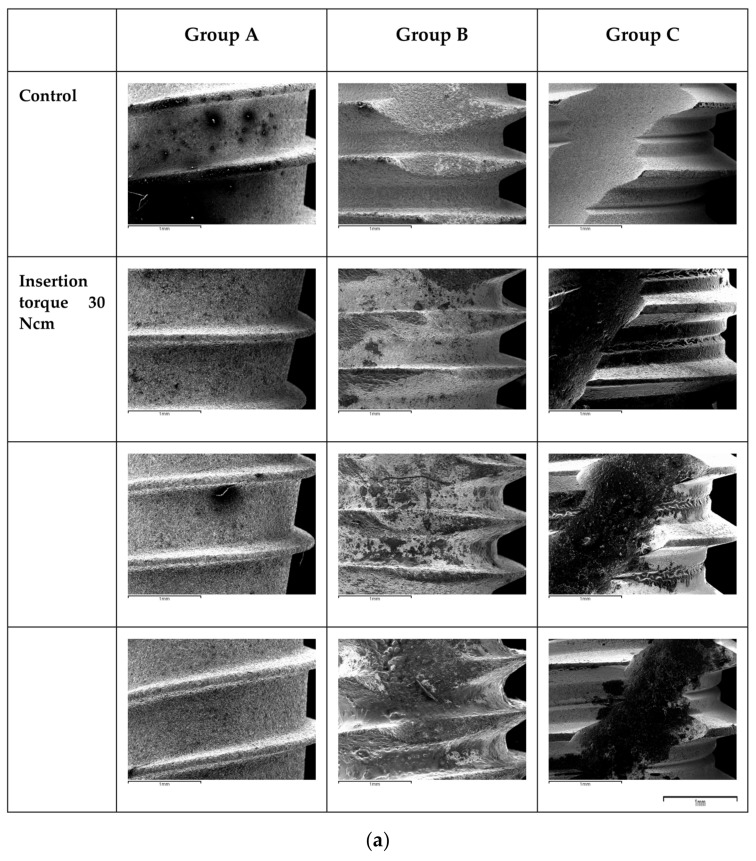

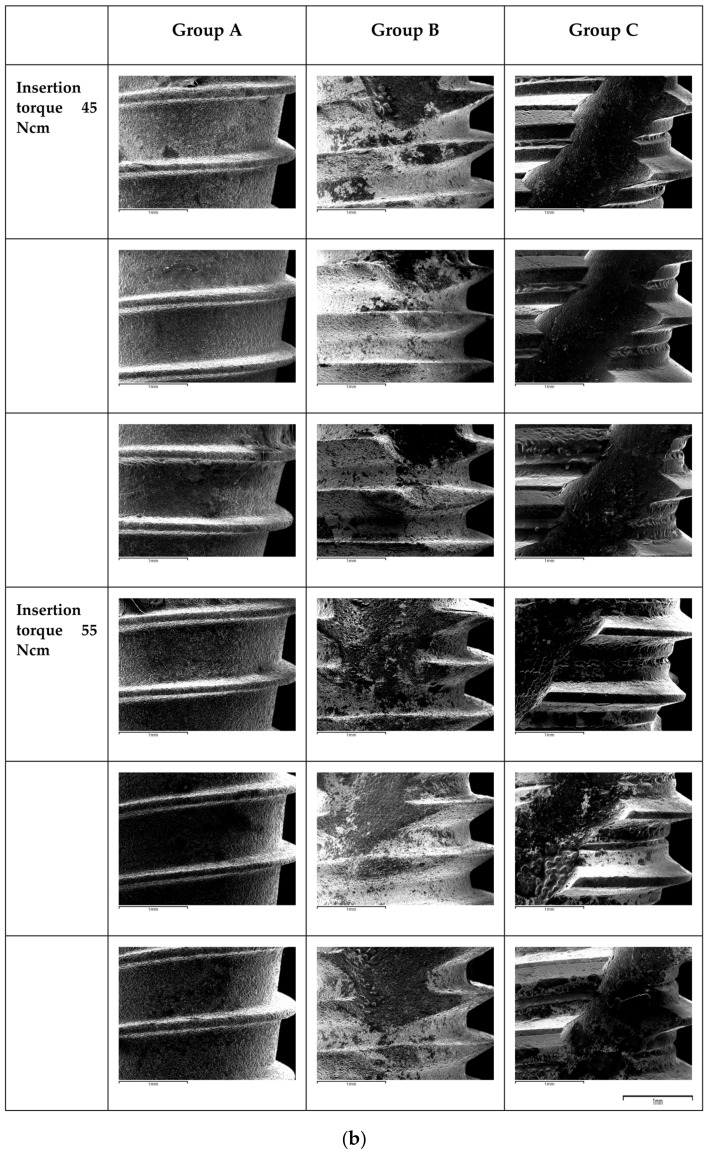
(**a**) SEM images (50×)of the apical third samples of each group are shown after the application of the predetermined insertion torque values. The images are of the 9 implants (Groups A, B and C) with their respective torques at 30, 45 and 55 Ncm. (**b**) SEM images (50×) of the third apical samples of each group are shown after the application of the predetermined insertion torque values. The images are of the 9 implants (Groups A, B and C) with their respective torques at 30, 45 and 55 Ncm.

**Figure 5:**
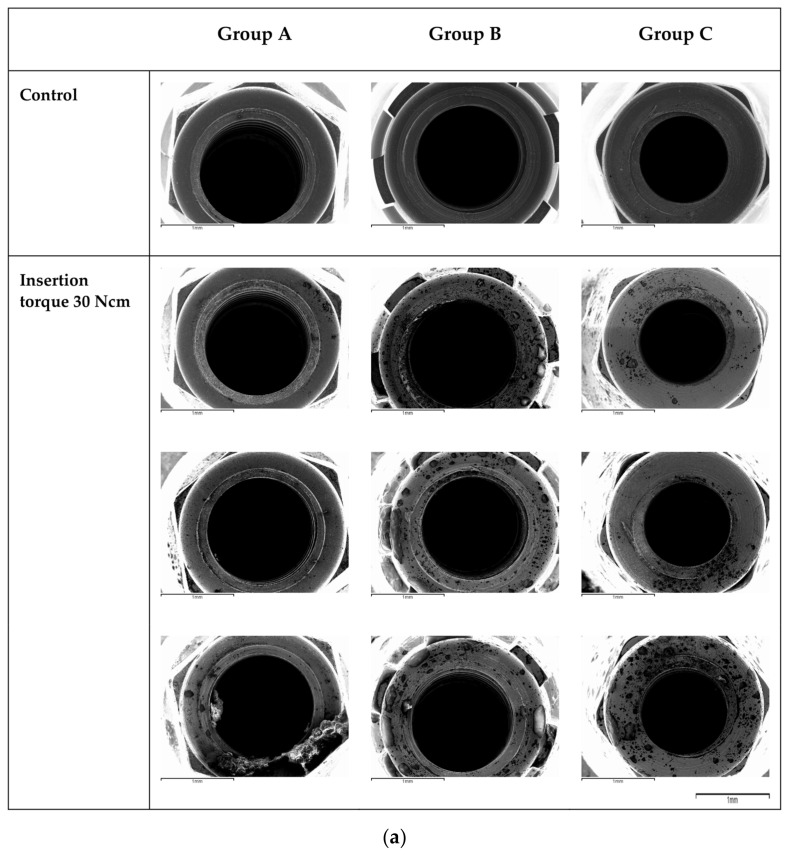

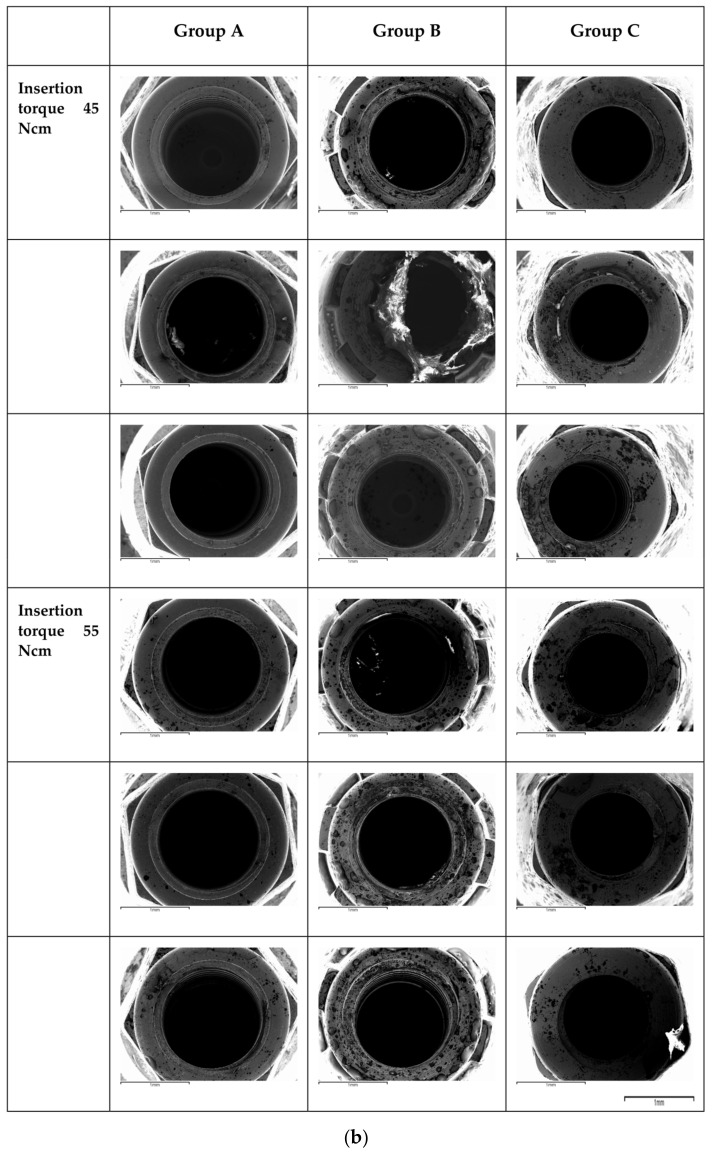
(**a**) SEM images (50×) of the connection samples of each group are shown after the application of the predetermined insertion torque values. The images are of the 9 implants (Groups A, B and C) with their respective torques at 30, 45 and 55 Ncm. (**b**) SEM images (50×) of the connection samples of each group are shown after the application of the predetermined insertion torque values. The images are of the 9 implants (Groups A, B and C) with their respective torques at 30, 45 and 55 Ncm.

Independently of the implant group, the alterations were localized at the more apical part, where threads were randomly broken during insertion. Furthermore, on the thread crests, topographical changes such as surface leveling and abrasion facets were seen (Figure 4a,b and Figure 5a,b).

## 4. Discussion

This ex vivo study revealed the impact of increasing insertion torques on the implant surface and connection macro- and micro-geometries. Surface alterations were shown on the apical part of the implants, as well as on the implant connections. Increasing insertion torques lead to increasing surface alterations.

Traditionally, it was widely accepted that implant interface failure was a result of bone resorption caused by an excessive load instead of the micromotion of the implant during the healing period. In contrast to this assumption, a number of studies have been carried out where both the displacement of the implant and the load were controlled, and it was evident that the resorption was caused by the implant’s instability, even when only minor stresses were applied. These investigations demonstrated that in cortical bone, resorption of the bone surface could be caused by a displacement of just a few micrometers at the bone interface. This resorption process widened the space between the movable surfaces, which put a strain on regenerating tissues. The fundamental premise of the “strain” theory is that nearly no movement should be permitted between the implant and the surrounding bone when they are strongly compressed, leaving just a small gap between them. Otherwise, even tiny motions could cause strain, which would destroy the cells and blood vessels that were developing in the gap [5,7]. Therefore, it is suggested that implant prognosis improves when the clinician achieves high insertion torques in surgical protocols [3,4,5]. In fact, an experimental animal study revealed a micromotion threshold between 50 and 100 mm, above which micromotion causes bone resorption at the interface and results in fibrosis surrounding endosseous implants [20,21,22]. On the contrary, other studies have suggested that a high insertion torque produces compressive forces in the peri-implant bone, which may alter the bone microcirculation and produce bone necrosis and therefore compromise osseointegration [18]. Additionally, the compressive forces can lead to mechanical complications [23], such as deformation of the prosthetic connection, abrasion of the threads, or biological complications, such as peri-implantitis, due to the release of titanium particles in the surrounding bone [24]. In fact, a similar ex vivo study showed that implant placement could release up to 0.5 mg of titanium particles at the bone/implant interface [25]. Furthermore, regarding the deformation at the level of the implant connection, the study of Gehrke et al. reported that independently of the type of implant/abutment connection, torque values exceeding 80 Ncm resulted in a significant irreversible deformation, which could lead to clinical complications, such as abutment and crown loosening, screw fractures, abutment adaptation issues and bacterial microfiltration [26,27]. However, in the latter study, the torque application was performed on the implant completely static, which is not similar to any clinical scenario [23]. In contrast, in our study, implants were in motion during insertion into bony tissue, which in its turn could absorb some of the applied stress. Moreover, the microscopic analysis performed was with an optical microscope, in contrast to our study, where the SEM microscope permitted higher magnification and resolution. Consequently, this last feature could highlight very small changes at the level of the implant surface and connection, which makes the nature of the study highly innovative.

### 4.1. Surface Damage at the Level of the Apical Third

The findings in this study demonstrated that, independently from the design, implants presented remarkable changes at the level of the threads and in the apical third. These results are in agreement with the study conducted by Mints et al. [24], where SEM was used to compare anodized and sandblasted implant surfaces before and after insertion. Anodized implants demonstrated the most significant damage associated with insertion; the entire porous oxide layer was removed at the apical region and on the crests of the threads, while the neck area was totally preserved. Senna et al. [25] also described microscopically observable alterations using SEM and interferometry to analyze three distinct implants with varying surface topographies following insertion in fresh cow rib bone blocks. They found that following insertion, those surfaces linked with more convex structures had more damage and a more pronounced reduction in material volume.

### 4.2. Surface Damage at the Level of the Connection

The goal of this study was also to examine the deformation of implant connections subjected to different insertion torques. There are several types of prosthetic connections; the most commonly used are internal and external hexagonal connections. Implants with an internal hexagonal connection have been successfully used because they prevent rotation of the abutment [10]. The degree of rotational freedom between the implant and the prosthetic abutment is a key aspect in determining the long-term stability of the two components (implant and abutment), and increased rotational mobility has been linked to a higher rate of screw loosening. In our study (in which internal hexagonal and conical connections were used), it was observed that from the application of 45 and 55 Ncm, deformations were observed in the prosthetic connection. As seen in the SEM images, the deformation was proportional to the insertion torque value. This emphasizes the importance of avoiding the use of excessive torque (>45 Ncm) when placing implants. In fact, the deformation of the prosthetic connection could result in an increased gap at the implant/abutment interface, allowing bacteria to filtrate and subsequently leading to marginal bone loss. Nevertheless, these insertion torque threshold values are in contrast with the study carried out by Teixeira et al., who assessed the deformation of several platforms subjected to a manual torsion test of 80 and 120 Ncm [28]. In the study, external hexagonal, internal hexagonal, and morse taper connections were evaluated. Their results showed that the morse and the internal hexagonal connection demonstrated the least deformation when an insertion torque of 120 Ncm was applied. All groups presented alterations of the implant microgeometry with a torque of 80 Ncm, a higher threshold value compared with the one found in our study (45 Ncm). Once again, the assessment of the deformations was performed with an optical microscope in Teixeira’s study, in contrast to the SEM microscope employed in our study. This could have underestimated the alterations at the level of the connection with lower torque values, which could have been potentially detected with a higher resolution and magnification SEM microscope.

### 4.3. Impact of the Surface Damage: Titanium Release

During implant placement, localized areas of stress concentration occur on the implant surface, which could undermine the integrity of the implant, and as a result, cause a release of titanium particles in the surrounding bone [25]. The presence of titanium debris could result in inflammation and subsequent bone resorption. This was evidenced by Mints’ study [24]. In fact, it was shown that implant insertion could release up to 0.5 mg of particles at the implant-bone interface [25]. Furthermore, prior research found that 0.2–3.0 mg of loose titanium particles could cause aseptic osteolysis, resulting in significant and irregular osteoclastic activity and resorbed bone areas that were 8% to 35% higher than the control sites at 7 to 10 days, respectively [29,30]. A persistent inflammatory response has been found to be one of the primary causes of the aseptic loosening of implants when wear particles are present. Inflammatory cytokines such as IL-6, IL-8 and tumor necrosis factor alpha have been demonstrated to be enhanced by titanium particles [24]. As a result, loss of integrity of the implant surface can have a negative impact on the healing of the surgical site.

### 4.4. Impact of the Material Properties

Since Groups A, B and C are of different titanium compositions, it could be speculated that titanium grade 5 (Group A) is the grade that tolerates best the torsional forces during implant placement, compared to grade 23 (Group B) and grade 4 (Group C). In fact, because it contains less iron and the interstitial elements carbon and oxygen, titanium grade 23 6AL-4V ELI (Extra-Low Interstitial) is said to be purer than 6AL-4V Grade 5, and in consequence, less resistant, which could explain the results obtained in our study. Furthermore, the titanium grade employed in Group C is a novel composition of titanium called grade 4 achieved with the cold forming method, which is described to enhance the mechanical properties of the grade 4 titanium. However, according to our study, grade 5 alloy was revealed to have superior mechanical properties, shown by the minimal sustained surface damage compared to grade 23 titanium alloy and grade 4 (cold forming method) titanium.

### 4.5. Limitations of the Study

The main limitation was the nature of the study being in vitro. Moreover, the observational nature of the study led to subjective evaluations of the deformities; consequently, it was not possible to know if surface alterations were clinically relevant. Furthermore, the current findings are not applicable to implants with different macro- and micro-geometries than the ones that were evaluated. Finally, no attempt to quantify the size of the deformities was made; therefore, statistics could not be performed, and purely descriptive work was carried out.

## 5. Conclusions

Within the limitations of this ex vivo study, the findings revealed that high torque values cause implant surface deformations. When compared to the crestal area, more damage was found at the apical portion of the implants and at the level of the implant threads. The alterations sustained by the implants were proportional to the insertion torque intensities; 45 Ncm and higher insertion torques resulted in more significant damage at both the implant surface and the connection. The Group A implant surface appeared to be the most resistant to high insertion torques.

## Data Availability

The data that support the findings of this study are available from the corresponding author upon reasonable request.
